# Molecular identification of tick-borne pathogens in asymptomatic individuals with human immunodeficiency virus type 1 (HIV-1) infection: a retrospective study

**DOI:** 10.1186/s12879-018-3140-7

**Published:** 2018-05-18

**Authors:** Renata Welc-Falęciak, Justyna D. Kowalska, Małgorzata Bednarska, Magdalena Szatan, Agnieszka Pawełczyk

**Affiliations:** 1 Department of Parasitology, Faculty of Biology, University of Warsaw, 1 Miecznikowa Street, 02-096 Warsaw, Poland; 2 Department of Adults’ Infectious Diseases, Medical University of Warsaw, 37 Wolska Street, 01-201 Warsaw, Poland; 3 Department of Immunopathology of Infectious and Parasitic Diseases, Medical University of Warsaw, 3C Pawińskiego Street, 02-106 Warsaw, Poland; 4 AmerLab Ltd. Diagnostic Laboratory of Parasitic Diseases and Zoonotic Infections, Biological and Chemical Research Centre, 101 Żwirki and Wigury Street, 02-089 Warsaw, Poland

**Keywords:** HIV, Tick-borne disease, Human granulocytic anaplasmosis, Lyme borreliosis

## Abstract

**Background:**

The studies on the occurrence and diversity of tick-borne infections in HIV-infected individuals have been few, and the subject has been relatively neglected when compared with other common infections associated with HIV. In HIV-positive patients in whom a serological diagnostics is complicated due to reduced positive predictive value, a method where the microorganism is detected directly is of great value. Therefore, we performed a molecular study to ascertain the prevalence and incidence of tick-borne infections in HIV-infected persons in Poland, an endemic area for *Ixodes ricinus* ticks.

**Methods:**

Genomic DNA was isolated from whole blood of tested patients. Detection of tick-borne pathogens was performed by amplification and sequencing of different loci. Molecular and phylogenetic analyses of obtained nucleotide sequences were performed. Serum samples were analyzed for antibodies against tick-borne pathogens by using commercial tests in all patients.

**Results:**

Among 148 studied blood samples from HIV-infected patients, two cases (1.4%) of infection with tick-borne pathogen were reported. No symptoms of tick-borne infection were observed in these cases. In one case a patient was infected with *Anaplasma phagocytophilum* – the agent of human granulocytic anaplasmosis (HGA) and in the other with *Borrelia garinii*.

**Conclusions:**

Our study revealed the first case of HIV positive patient infected with *A. phagocytophilum.* Asymptomatic tick-borne infection can occur in HIV-positive patients. The detailed history of tick bites, especially in endemic tick areas, should be considered as part of anamnesis in routine clinical care of HIV-positive patients.

## Background

In patients diagnosed with HIV-1 (human immunodeficiency virus type 1), immunodeficiency significantly increases the risk of disease caused by pathogens that expand as a consequence of reduced level of T lymphocyte (LT) CD4 + cells, and pathogenicity is usually controlled by humoral and cellular immune responses [1]. However, since the introduction of antiretroviral drugs and effective regimens of antiretroviral therapy (highly active antiretroviral therapy – HAART), the prognosis for humans infected with HIV-1 has been significantly improved, thereby enabling them to lead active lives with outdoor activities that increase the risk of tick infestation [2].

Pathogens that cause tick-borne diseases (TBDs) constitute a significant problem for the public health. The observed increase of tick count, their high activity in the natural environment and in the urban area increases the risk of tick-borne infections [3–5]. In Europe, *Ixodes ricinus* is the most prevalent and widely distributed tick species which serves as the most important vector for several microbial pathogens, including *Borrelia, Anaplasma, Babesia* and *Rickettsia* [6]. Additionally, new tick-borne pathogens, *Candidatus* Neoehrlichia mikurensis (CNM) and *Borrelia myiamotoi*, have been observed in ticks as well as in humans in Europe, e.g. Poland [7–9]. So far in Europe, from the wide range of tick-borne pathogens, only *Borrelia*, *Babesia* and *Rickettsia* infections have been detected in HIV-infected humans [10–14].

*Borrelia burgdorferi* spirochetes comprise the etiological agent of Lyme disease, the most common tick-borne disease in the USA and Europe [15]. The most common clinical symptom of the early phase of infection is *erythema migrans* (EM). In the course of disseminated infection, spirochetes penetrate into body tissues, especially skin, central nervous system, joints and heart. *Borrelia burgdorferi* sensu stricto is the sole causative agent of Lyme disease in North America [15]. In Europe, at least five species are considered to be pathogenic for humans (*B. afzelii*, *B. garinii*, *B burgdorferi*, *B. spielmanii*, *B. bavariensis*), resulting in much higher than in the United States of America variety of clinical symptoms of Lyme disease [15]. Borreliosis is a rare co-infection in HIV-positive patients, and till now, it has only few cases have been reported [10–12, 16–18]. Most cases were diagnosed as early infections, and neuroborreliosis has been confirmed in patients from the Netherlands and Sweden [11, 12]. Until now it remains unclear whether HIV-positive persons are at increased risk of acquiring or atypical course of tick-born infections and studies in this area are scarce. However it could be expected that HIV-1 and *Borrelia* co-infection is more common than it has been described so far [12]. Diagnosing of Lyme disease using serology- based methods in HIV-positive patients is believed to be complicated due to their reduced positive predictive value of serology [19]. Currently, thanks to the improvement of the effectiveness of treatment, the majority of HIV positive patients has a significant improvement in the immune system function and consequently the positive predictive value (PPV) risk for serological tests is significantly lower. Moreover, false positive serologic findings are described in patients with neurological infections with other spirochetes such as *Treponema pallidum* [20].

Babesiosis is a tick-borne disease caused in humans by protozoa of the *Babesia* genus, mainly *B. microti* in the USA and *B. divergens* in Europe (isolated cases of infection with *B. microti* and *B. venetorum* have been noted). Furthermore, transfusion-transmitted babesiosis has been recognized, and a few congenital human cases due to *B. microti* have been documented [21–23]. Babesiosis in HIV-infected individuals was observed to date in the United States of America [24–29], and one case was noted in Europe (Spain; [13]). Molecular studies have confirmed infection with *B. microti* (the USA; [28, 29]) or *B. divergens* (Spain, [13]). In a few instances, babesiosis was observed either in asplenic, HIV-positive patients [25, 26], or its symptoms appeared only after splenectomy [13]. In HIV-infected humans a long-term chronic phase of infection lasting several months despite treatment was observed with relapses which required blood transfusions because of the high parasitemia (8.5% of infected red blood cells, iRBC; [29]).

Tick-borne rickettsiosis is caused by intracellular bacteria belonging to the spotted fever group (SFG) of the Rickettsiaceae family. The main clinical manifestations of a rickettsial syndrome in humans are fever, rash, and eschar at the site of the tick bite (‘tache noire’) [30]. Isolated cases of Mediterranean spotted fever (MSF), caused mainly by *R. conorii* and *R. monacensis*, have been reported in HIV-infected humans [14, 31]. In some cases, primary HIV infection can mimic MSF presentation [31].

The studies on the occurrence and diversity of tick-borne infections in HIV-infected individuals have been few, and the subject has been relatively neglected when compared with other infections associated with HIV. Non-specific symptoms of tick-borne diseases poses a challenge in clinical care and may lead to misdiagnosis, especially in HIV-positive patients, who often present with many clinical symptoms. Additionally, in immunocompromised patients, prolonged prepatent periods with a significant delay of antibody production may occur, and the results of a serological test may be misinterpreted [32, 33]. The purpose of this study was to investigate the prevalence and risk for tick-borne infection in HIV-1-infected individuals in Poland, where tick-borne diseases are endemic. To the best of our knowledge, this is the first molecular study on the occurrence of the most frequent pathogens transmitted by ticks (*Borrelia* spp., *Babesia* spp., *Rickettsia* spp., *A. phagocytophilum*, *Candidatus* Neoehrlichia spp.) in HIV-1-infected humans.

## Methods

### Blood samples/selection of patients

The retrospective study was conducted on HIV-positive patients who had no history of tick bite and no clinical manifestation characteristic for tick-borne diseases. In 2013, blood samples were collected from 148 patients routinely followed at the HIV Outpatients’ Clinic of the Hospital for Infectious Diseases in Warsaw. Written informed consent was obtained from all patients and the study protocol followed ethical guidelines of the 2013 Declaration of Helsinki. The study was approved by the Internal Review Board of the Warsaw Medical University (no. AKBE/24/16). All ethical approvals for the study have been obtained according to Polish regulations.

### DNA isolation and PCR amplification

Genomic DNA was isolated from whole blood using the DNeasy Blood and Tissue kit (Qiagen, Crawley, United Kingdom). Detection of tick-borne pathogens was performed by amplification and sequencing of different loci: (1) the flagellin gene (*flaB*) and the *16S rRNA* gene for *Borrelia* spp. [34, 35]; (2) the glycerophosphodiester phosphodiesterase (*glpQ*) gene for *B. myiamotoi* [36]; (3) the *18S rRNA* gene of *Babesia* spp. [37]; (4) the citrate synthase (*gltA*) gene for *Rickettsia* spp. [38]; (5) the *16S rRNA* gene and *groESL* heat shock operon for *A. phagocytophilum*, as well as Ca. *Neoehrlichia* spp. [39, 40]. Negative controls were performed in the absence of template DNA. *Anaplasma phagocytophilum*, Ca. Neoehrlichia mikurensis, *Borrelia afzelii*, *B. garinii* and *B. myiamotoi* DNA extracted from blood of positive patients diagnosed in AmerLab Ltd. Diagnostic Laboratory of Parasitic Diseases and Zoonotic Infections, *Babesia microti* King College strain DNA isolated from BALB/c mice blood and *Rickettsia helvetica* DNA obtained from infected tick were used as positive controls. Amplicons were visualized with Midori Green stain (Nippon Genetics Europe GmbH) following electrophoresis in 2% agarose gels. Amplicons were purified and sequenced by a private company (Genomed S.A., Poland) in both directions.

### Phylogenetic analysis

Obtained nucleotide sequences were analyzed using BLAST-NCBI and MEGA v. 7.0 software [41] for sequence alignment, species typing and phylogenetic relationships. After testing the data for the best substitution model, phylogenetic trees were obtained using Maximum Likelihood as the tree construction method and Tamura 92 + I parameter algorithm as a distance method. For comparison, sequences of *Anaplasma* spp. obtained from GenBank (www.ncbi.nlm.nih.gov) were implemented in the sequence alignment. The stability of inferred phylogenies was assessed by bootstrap analysis of 1000 randomly generated sample trees.

### Serological tests

All patient serum samples were analyzed for antibodies against tick-borne pathogens by using: (i) *Borrelia* IgM and IgG ELISA tests (Biomedica Laboratories, Vienna, Austria) (ii) Western Blot: *recom*Line *Borrelia* IgM and *recom*Line *Borrelia* IgG (Microgen, Neuried, Germany) for *Borrelia burgdorferi* s.l.; (iii) *Ehrlichia chaffeensis* and *Anaplasma phagocytophilum* IFA IgM Antibody Kit (Fuller Laboratories, California, the USA) for *Anaplasma phagocytophilum*; (iv) *Babesia microti* IFA IgM and IgG antibody kits (Fuller Laboratories, California, the USA) for *Babesia* spp.; (v) Spotted Fever *Rickettsia* IgG EIA antibody kit (Fuller Laboratories, California, the USA) for *Rickettsia* spp. with the manufacturer’s cut-off levels and interpretation criteria.

### New sequences

New nucleotide sequences have been deposited in GenBank with the accession numbers: MG570466 for *groESL* of *A. phgocytophilum* and MG570467 for *flaB* of *B. garinii*.

## Results

In total 148 blood samples from asymptomatic HIV-positive patients were included, predominantly male (140 patients, 95%) with mean age of 33 years. The median lymphocyte CD4+ T cell count was 465/μl with 19% of patients with less than 300/μl. Most of these patients (82%; *n* = 121) were on HAART.

Among the 148 studied blood samples two cases (1.4%) of infection with tick-borne pathogen were reported.

- Patient 1. A 31-year-old man from Georgia, diagnosed with HIV in 2011 and HCV in 2009. Laboratory tests showed elevated liver enzyme levels, plasma HIV RNA levels of 6938 copies/ml and a CD4+ T cell count of 534/μl. The patient was hospitalized in 2013 due to infection of the urinary tract and in 2014 because of heart arrhythmia not confirmed in ECG. The patient had no history of tick bite and no clinical manifestation characteristic for tick-borne diseases.

**Fig. 1:**
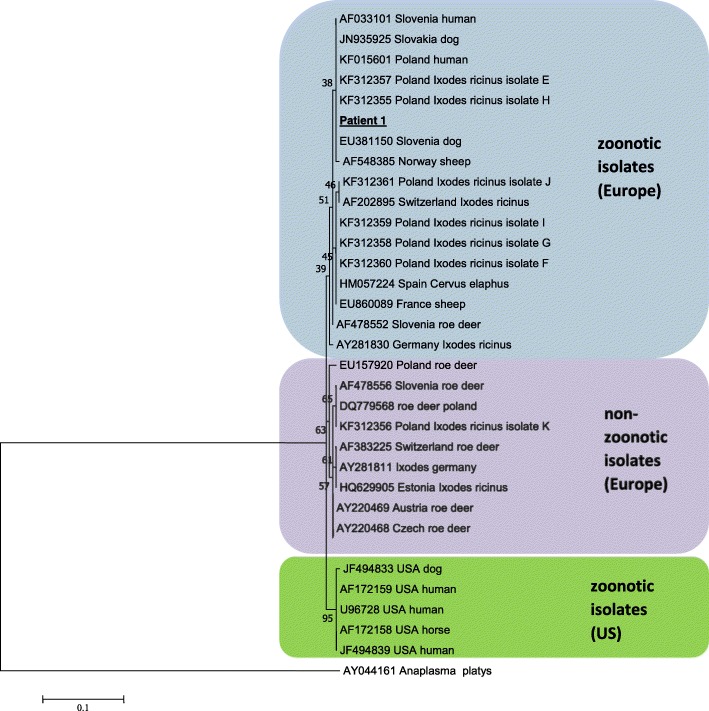
Phylogenetic tree of the *Anaplasma* isolate studied in the current work and chosen isolates from GenBank based on the fragment of the groESL heat shock operon. Numbers at the nodes of the tree indicate bootstrap values (1000 replicates)

The PCR analysis revealed the presence of *A. phagocytophilum-*specific 540 bp fragment of the *16S rRNA* gene and the 1200 bp fragment of the *groESL* heat shock operon. Sequencing analysis of the PCR products using both sets of primers showed a high level of homology (< 99.8%) with *A. phagocytophilum* strains pathogenic for humans and animals (Fig. 1). The tests revealed the presence of specific IgM and IgG antibodies against *A. phagocytophilum* using indirect immunofluorescence assay (IFA). The DNAs presence of *Borrelia* spp., *Babesia* spp., *Rickettsia* spp. and CNM was not confirmed. The results of a serological test for the above-mentioned tick-borne pathogens (excluding CNM and *B. myiamotoi*) were also negative.

- Patient 2. 25-year-old man, diagnosed with HIV in 2013. The patient had been using HAART since 2013. Laboratory tests showed lymphocyte CD4+ T cell count of 424/μl, plasma HIV RNA load was undetectable. Routine laboratory tests showed no signs of any infection. He was diagnosed and treated with syphilis twice. He had no history of tick bites, rash, erythema migrans or other signs of early or late-stage Lyme borreliosis.

A 357 bp fragment of *Borrelia 16S rRNA* gene was amplified from the patient’s blood by PCR. The partial *flaB* fragment (600 bp) was also amplified since the *16S rRNA* gene is too conserved for analysis of *Borrelia* genetic heterogeneity. Alignment and BLAST-NCBI analyses of *16S rRNA* and *flaB* gene fragments revealed the presence of *B. garinii* identical to isolates obtained from *Ixodes ricinus* in Poland (*flaB*: HM345898) and the Czech Republic (*flaB:* JN828685), as well as from ticks in Spain (*16S rRNA*: DQ147793) and Russia (*16S rRNA*: KY346891).

The results of serological tests (ELISA and WB) were ambiguous: for IgM antibodies, ELISA test was 13.5 BBU/ml (not confirmed by WB: Op.), for IgG antibodies the test results were indeterminate (ELISA: 9 BBU/ml; WB: 6p.). The DNAs presence of *A. phagocytophilum*, *Babesia* spp., *Rickettsia* spp. and CNM was not confirmed. The results of a serological test for the above-mentioned tick-borne pathogens (excluding CNM and *B. myiamotoi*) were also negative.

## Discussion

Till now studies investigating the epidemiology of tick-borne pathogen infection have focused on healthy individuals with normal immunological function and living in endemic regions or clinical findings among hospitalized patients. In HIV-positive patients, where a serological diagnosis is complicated due to reduced positive predictive value, a method where the microorganism detected directly might be of great value [11]. Hence, we conducted a retrospective, molecular study to ascertain the prevalence and incidence of tick-borne infections in HIV-infected persons with no clinical symptoms located in Poland, an endemic area for *I. ricinus* ticks. The diagnostic methods of tick-borne diseases used in this study are compatible with recommendation of Polish Society of Epidemiology and Infectious Diseases [42].

The results of our study revealed the first case of a patient co-infected with HIV and *A. phagocytophilum* – the agent of human granulocytic anaplasmosis (HGA). The patient had no history of tick bite or clinical manifestation of anaplasmosis. It is well known that the early symptoms of HGA are nonspecific, often mimicking a viral infection, as well as that almost a quarter of patients with proven HGA does not report exposure to ticks [43]. However, the clinical range of HGA spans from asymptomatic infections to fatal diseases. Most published case reports indicate that HGA is a mild, self-limited illness that resolves without antibiotic treatment [44]. Asymptomatic infection with *A. phagocytophilum* may occur frequently, as suggested in a seroepidemiologic study in which 30% of hunters in tick-endemic area seroconverted without symptoms [45]. Serious opportunistic infections can occur in immunocompromised patients during the course of HGA and fatal cases of herpes simplex or *Candida albicans* esophagitis, as well as invasive pulmonary aspergillosis, are described [46–48]. Most patients with HGA present with non-specific changes in routine hematological and chemistry blood tests, e.g. leucopenia, thrombocytopenia and mild to moderate evaluation of hepatic transaminase activities, which has been also noted in our patient 1. However, it is difficult to define clearly whether observed elevated liver enzyme levels is caused by HGA or HCV infection.

HGA can be laboratory-confirmed by examination of peripheral blood smear, PCR or isolation of *A. phagocytophilum* in HL-60 promyelocytic leukemia cell cultures inoculated with acute phase blood during an early stage of infection as well as by serological tests (IFA) (3–6 weeks after infection) which can be confirmed by PCR [49]. Our laboratory results of molecular and serological tests fulfilled the criteria for confirmation of anaplasmosis and suggested not acute stage of the illness. Nevertheless, *Anaplasma*-specific antibodies can persist for months or years in the absence of any clinical signs of infection. Thus, changes in antibody titers cannot be used as monitors of effective treatment or recovery [50, 51].

Molecular analyses have indicated that some strains/genetic variants of *A. phagocytophilum* that are pathogenic for humans and domestic animals, circulate widely in nature in different hosts and display different vector tropisms and degrees of host pathogenicity [8, 52, 53]. Previous studies have revealed two distinct genetic lineages of *A. phagocytophilum*: (1) genetic variants detected in humans, ticks, dogs, horses, sheep and red deer from Europe and USA which are believed to be pathogenic; and (2) genetic variants isolated from ticks and roe deer in Europe that are probably non-zoonotic strains (Fig. 1) [8, 52]. The results of our molecular and phylogenetic analysis strongly suggest that infection in this patient was acquired with pathogenic variant of *A. phagocytophilum* that is closely related to *A. phagocytophilum* strains isolated previously from human and ticks in Poland and belongs to zoonotic group of isolates known to be pathogenic for humans (Fig. 1) [8, 53]. Interestingly, our previous studies demonstrated that the occurrence of zoonotic species/strains of *A. phagocythophilum* in *I. ricinus* ticks are mainly restricted to urban areas compared to natural habitants where tick abundance was significantly higher [8].

Despite the high incidence of HIV, co-infection with *B. burgdorferi* is rarely reported in medical journals [11, 12]. The vast majority of cases involve typical Lyme neuroboreliosis symptoms, such as meningoradiculitis and facial palsy [12]. Little is known about the course of Lyme neuroboreliosis in HIV patients, and the mechanism by which impaired immunity in HIV infection might influence the course of disease in Lyme neuroboreliosis still needs to be clarified. Animal models have shown that lymphocytes T CD4+ facilitate clearance of *B. burgdorferi* [54] and immunodeficiency leads to higher spirochete burdens and higher infectivity of these bacteria [55]. In HIV positive patients, the relatively higher HIV viral load in cerebrospinal fluid compared with plasma in the course of *B. burgdorferi* infection was observed [12]. Our patient had no signs of early or late-stage Lyme borreliosis. However, *Borrelia garini* infection, the main agent of neuroborreliosis in Europe [56], was confirmed by a positive PCR for the *16S rRNA* and *flaB* gene fragment. In our patient, the antibiotic treatment of syphilis could have a positive effect on *B. burgdorferi* infection.

There is a well-known cross-reactivity between the *Borrelia* and *Treponema* spirochetes and false positive serologic findings are described in patients with neurological infections with other spirochetes such as *T. pallidum* [12, 20]. In our case, the results of a serological test were indeterminate. Patients with syphilis and borreliosis may present with identical clinical symptoms. Therefore, clinicians frequently rely on serological criteria to differentiate between the two [57]. The frequency of antibody detection in serum depends on the type of antigen used in the diagnostic test [58]. *B. burgdorferi* s.l. and *T. pallidum* are spirochaetes which share antigens, such as the 41-kDa flagellin and the 60-kDa bacterial antigen. Anti-flagellin serum antibody can be detected in 87% of patients infected by *B. burgdorferi* and 75% of patients infected by *T. pallidum*. The frequency of antibody detection in the serum to the 60-kDa antigen is 58 and 42.9% for borreliosis and syphilis respectively [58]. The flagellin is highly immunogenic and elicits the earliest detectable immune response after infection. This antigen’s fractions of *B. burgdorferi* are often used in Lyme antibody assays to improve the sensitivity of tests [58]. Nevertheless, these are false positive results in patients with syphilis [20, 57]. Accordingly, our data suggest that in patients with indeterminate Lyme screening and negative or indeterminate confirmatory testing, performance of PCR might be considered.

## Conclusions

In this study, we presented two cases of tick-borne infection in HIV-positive patients. In both cases, the diagnoses were based on molecular and serological tests. No symptoms of tick-borne infection in the cases could be explained by antibiotic therapy recommended due to urinary tract infection (patient 1) or *Treponema palladium* infection (patient 2). Asymptomatic tick-borne infection can occur in HIV-positive patients. The detailed history of tick bites, especially in endemic tick areas, should be considered as part of anamnesis in routine clinical care of HIV-positive patients.

## Abbreviations

CNM: *Candidatus* Neoehrlichia mikurensis
ECG: Electrocardiography
ELISA: Enzyme-linked immunosorbent assay
EM: *Erythema migrans*
HAART: Highly active antiretroviral therapy
HCV: Hepatitis C virus
HGA: Human granulocytic anaplasmosis
HIV-1: Human immunodeficiency virus type 1
IFA: Immunofluorescent assay
iRBC: Infected red blood cells
LT: T lymphocyte
MSF: Mediterranean spotted fever
PPV: Positive predictive value
SFG: Spotted fever group
TBDs: Tick-borne diseases
WB: Western blot

## References

[1] Chang CC, Crane M, Zhou J, Mina M, Post JJ, Cameron BA, Lloyd AR, Jaworowski A, French MA, Lewin SR (2013). HIV and co-infections. Immunol Rev.

[2] van Sighem AI, Gras LA, Reiss P, Brinkman K, de Wolf F (2010). ATHENA national observational cohort study. Life expectancy of recently diagnosed asymptomatic HIV-infected patients approaches that of uninfected individuals. AIDS.

[3] Daniel M, Materna J, Honig V, Metelka L, Danielová V, Harcarik J, Kliegrová S, Grubhoffer L (2009). Vertical distribution of the tick Ixodes ricinus and tick-borne pathogens in the northern Moravian mountains correlated with climate warming (Jeseníky Mts., Czech Republic). Cent Eur J Public Health.

[4] S J, Viljugrein H, Hofshagen M, Brun-Hansen H, Kristoffersen AB, Nygård K, Brun E, Ottesen P, Sævik BK, Ytrehus B (2011). Multi-source analysis reveals latitudinal and altitudinal shifts in range of Ixodes ricinus at its northern distribution limit. Parasit Vectors.

[5] Jaenson TG, Jaenson DG, Eisen L, Petersson E, Lindgren E (2012). Changes in the geographical distribution and abundance of the tick Ixodes ricinus during the past 30 years in Sweden. Parasit Vectors.

[6] Rizzoli A, Silaghi C, Obiegala A, Rudolf I, Hubálek Z, Földvári G, Plantard O, Vayssier-Taussat M, Bonnet S, Spitalská E, Kazimírová M (2014). Ixodes ricinus and its transmitted pathogens in urban and Peri-urban areas in Europe: new hazards and relevance for public health. Front Public Health.

[7] Welc-Falęciak R, Siński E, Kowalec M, Zajkowska J, Pancewicz SA (2014). Asymptomatic "Candidatus Neoehrlichia mikurensis" infections in immunocompetent humans. J Clin Microbiol.

[8] Welc-Falęciak R, Kowalec M, Karbowiak G, Bajer A, Behnke JM, Siński E (2014). Rickettsiaceae and Anaplasmataceae infections in *Ixodes ricinus* ticks from urban and natural forested areas of Poland. Parasit Vectors.

[9] Kowalec M, Szewczyk T, Welc-Falęciak R, Siński E, Karbowiak G, Bajer A (2017). Ticks and the city - are there any differences between city parks and natural forests in terms of tick abundance and prevalence of spirochaetes?. Parasit Vectors.

[10] R C, Machala L, Bojar M, Rozsypal H, Pícha D (2006). Neuroborreliosis in an HIV-1 positive patient. Infection.

[11] van Burgel ND, Oosterloo M, Kroon FP, van Dam AP (2010). Severe course of Lyme neuroborreliosis in an HIV-1 positive patient; case report and review of the literature. BMC Neurol.

[12] Bremell D, Säll C, Gisslén M, Hagberg L (2011). Lyme neuroborreliosis in HIV-1 positive men successfully treated with oral doxycycline: a case series and literature review. J Med Case Rep.

[13] González LM, Castro E, Lobo CA, Richart A, Ramiro R, González-Camacho F, Luque D, Velasco AC, Montero E (2015). First report of Babesia divergens infection in an HIV patient. Int J Infect Dis.

[14] Colomba C, Siracusa L, Madonia S, Saporito L, Bonura C, De Grazia S, Giammanco GM (2013). A case of spotted fever rickettsiosis in a human immunodeficiency virus-positive patient. J Med Microbiol.

[15] Stanek G, Wormser GP, Gray J, Strle F (2012). Lyme borreliosis. Lancet.

[16] Garcia-Monco JC, Frey HM, Villar BF, Golightly MG, Benach JL (1989). Lyme disease concurrent with human immunodeficiency virus infection. Am J Med.

[17] Dudle G, Opravil M, Lüthy R, Weber R (1997). Meningitis after acute *Borrelia burgdorferi* infection in HIV infection. Dtsch Med Wochenschr.

[18] Cordoliani F, Vignon-Pennamen MD, Assous MV, Vabres P, Dronne P, Rybojad M, Morel P (1997). Atypical Lyme borreliosis in an HIV-infected man. Br J Dermatol.

[19] Raoult D, Hechemy KE, Baranton G (1989). Cross-reaction with Borrelia burgdorferi antigen of sera from patients with human immunodeficiency virus infection, syphilis, and leptospirosis. J Clin Microbiol.

[20] Blatz R, Kühn HJ, Hermann W, Rytter M, Neurosyphilis RAC (2005). Neuroborreliosis. Retrospective evaluation of 22 cases. Nervenarzt.

[21] Joseph JT, Purtill K, Wong SJ, Munoz J, Teal A, Madison-Antenucci S, Horowitz HW, Aguero-Rosenfeld ME, Moore JM, Abramowsky C, Wormser GP (2012). Vertical transmission of *Babesia microti*, United States of America. Emerg Infect Dis.

[22] Aderinboye O, Syed SS (2010). Congenital babesiosis in a four-week-old female infant. Pediatr Infect Dis J.

[23] Leiby DA (2011). Transfusion-transmitted Babesia spp.: bull's-eye on Babesia microti. Clin Microbiol Rev.

[24] Benezra D, Brown AE, Polsky B, Gold JW, Armstrong D (1987). Babesiosis and infection with human immunodeficiency virus (HIV). Ann Intern Med.

[25] Ong KR, Stavropoulos C, Inada Y (1990). Babesiosis, asplenia, and AIDS. Lancet.

[26] Machtinger L, Telford SR, Inducil C, Klapper E, Pepkowitz SH, Goldfinger D (1993). Treatment of babesiosis by red blood cell exchange in an HIV-positive, splenectomized patient. J Clin Apher.

[27] Falagas ME, Klempner MS (1996). Babesiosis in patients with AIDS: a chronic infection presenting as fever of unknown origin. Clin Infect Dis.

[28] Froberg MK, Dannen D, Bakken JS (2004). Babesiosis and HIV. Lancet.

[29] Vyas JM, Telford SR, Robbins GK (2007). Treatment of refractory *Babesia microti* infection with atovaquone-proguanil in an HIV-infected patient: case report. Clin Infect Dis.

[30] Faccini-Martínez ÁA, García-Álvarez L, Hidalgo M, Oteo JA (2014). Syndromic classification of rickettsioses: an approach for clinical practice. Int J Infect Dis.

[31] Segura F, Antón E, Font B, Sala M, Cervantes M (2002). Primary HIV type-1 infection misdiagnosed as Mediterranean spotted fever. Eur J Clin Microbiol Infect Dis.

[32] Moir S, Fauci AS (2017). B-cell responses to HIV infection. Immunol Rev.

[33] Pallikkuth S, L d A, S R, S P (2017). T follicular helper cells and B cell dysfunction in aging and HIV-1 infection. Front Immunol.

[34] Wodecka B, Leońska A, Skotarczak B (2010). A comparative analysis of molecular markers for the detection and identification of Borrelia spirochaetes in Ixodes ricinus. J Med Microbiol.

[35] Marconi RT, Garon CF (1992). Development of polymerase chain reaction primer sets for diagnosis of Lyme disease and for species-specific identification of Lyme disease isolates by 16S rRNA signature nucleotide analysis. J Clin Microbiol.

[36] Wagemakers A, Jahfari S, de Wever B, Spanjaard L, Starink MV, de Vries HJ, Sprong H, Hovius JW (2017). Borrelia miyamotoi in vectors and hosts in the Netherlands. Ticks Tick Borne Dis.

[37] Bonnet S, Jouglin M, L'Hostis M, Chauvin A (2007). Babesia sp. EU1 from roe deer and transmission within Ixodes ricinus. Emerg Infect Dis.

[38] Roux V, Rydkina E, Eremeeva M, Raoult D (1997). Citrate synthase gene comparison, a new tool for phylogenetic analysis, and its application for the rickettsiae. Int J Syst Bacteriol.

[39] Massung RF, Slater K, Owens JH, Nicholson WL, Mather TN, Solberg VB, Olson JG, Nested PCR (1998). Assay for detection of granulocytic ehrlichiae. J Clin Microbiol.

[40] Sumner JW, Nicholson WL, Massung RFPCR (1997). Amplification and comparison of nucleotide sequences from the groESL heat shock operon of Ehrlichia species. J Clin Microbiol.

[41] Kumar S, Stecher G, Tamura K (2016). MEGA7: molecular evolutionary genetics analysis version 7.0 for bigger datasets. Mol Biol Evol.

[42] Pancewicz SA, Garlicki AM, Moniuszko-Malinowska A, Zajkowska J, Kondrusik M, Grygorczuk S, Czupryna P, Dunaj J (2015). Polish Society of Epidemiology and Infectious Diseases. Diagnosis and treatment of tick-borne diseases recommendations of the polish Society of Epidemiology and Infectious Diseases. Przegl Epidemiol.

[43] Bakken JS, Dumler JS (2015). Human granulocytic anaplasmosis. Infect Dis Clin N Am.

[44] Ramsey AH, Belongia EA, Gale CM, Davis JP (2002). Outcomes of treated human granulocytic ehrlichiosis cases. Emerg Infect Dis.

[45] Tokarska-Rodak M, Plewik D, Michalski AJ, Kołodziej M, Mełgieś A, Pańczuk A, Konon H, Niemcewicz M (2016). Serological surveillance of vector-borne and zoonotic diseases among hunters in eastern Poland. J Vector Borne Dis.

[46] Hardalo CJ, Quagliarello V, Dumler JS (1995). Human granulocytic ehrlichiosis in Connecticut: report of a fatal case. Clin Infect Dis.

[47] Bakken JS, Krueth J, Wilson-Nordskog C, Tilden RL, Asanovich K, Dumler JS (1996). Clinical and laboratory characteristics of human granulocytic ehrlichiosis. JAMA.

[48] Jahangir A, Kolbert C, Edwards W, Mitchell P, Dumler JS, Persing DH (1998). Fatal pancarditis associated with human granulocytic Ehrlichiosis in a 44-year-old man. Clin Infect Dis.

[49] Ismail N, Bloch KC, McBride JW (2010). Human ehrlichiosis and anaplasmosis. Clin Lab Med.

[50] Aguero-Rosenfeld ME, Donnarumma L, Zentmaier L, Jacob J, Frey M, Noto R, Carbonaro CA, Wormser GP (2002). Seroprevalence of antibodies that react with *Anaplasma phagocytophila*, the agent of human granulocytic ehrlichiosis, in different populations in Westchester County, New York. J Clin Microbiol.

[51] Bakken JS, Haller I, Riddell D, Walls JJ, Dumler JS (2002). The serological response of patients infected with the agent of human granulocytic ehrlichiosis. Clin Infect Dis.

[52] von Loewenich FD, Baumgarten BU, Schröppel K, Geissdörfer W, Röllinghoff M, Bogdan C (2003). High diversity of ankA sequences of *Anaplasma phagocytophilum* among *Ixodes ricinus* ticks in Germany. J Clin Microbiol.

[53] Welc-Falęciak R, Kowalec M, Zajkowska J, Pancewicz SA, Siński E (2015). Clinical and molecular features of one case of human infection with *Anaplasma phagocytophilum* from Podlaskie Province in eastern Poland. Ann Agric Environ Med.

[54] Bockenstedt LK, Kang I, Chang C, Persing D, Hayday A, Barthold SW (2001). CD4+ T helper 1 cells facilitate regression of murine Lyme carditis. Infect Immun.

[55] Barthold SW, Sidman CL, Smith AL (1992). Lyme borreliosis in genetically resistant and susceptible mice with severe combined immunodeficiency. Am J Trop Med Hyg.

[56] Halperin JJ (2017). Neuroborreliosis. J Neurol.

[57] Naesens R, Vermeiren S, Van Schaeren J, Jeurissen A (2011). False positive Lyme serology due to syphilis: report of 6 cases and review of the literature. Acta Clin Belg.

[58] Aguero-Rosenfeld ME (2008). Lyme disease: laboratory issues. Infect Dis Clin N Am.

